# The RITE of Passage: Learning Styles and Residency In-Service Training Examination (RITE) Scores

**DOI:** 10.7759/cureus.12442

**Published:** 2021-01-03

**Authors:** Brenda G Fahy, Jean E Cibula, Lou Ann Cooper, Samsun Lampotang, Nikolaus Gravenstein, Terrie Vasilopoulos

**Affiliations:** 1 Anesthesiology, University of Florida College of Medicine, Gainesville, USA; 2 Neurology, University of Florida College of Medicine, Gainesville, USA; 3 Office for Educational Affairs, University of Florida College of Medicine, Gainesville, USA; 4 Anesthesiology/Orthopedics and Rehabilitation, University of Florida College of Medicine, Gainesville, USA

**Keywords:** educational models, learning styles, learning, curriculum, graduate medical education

## Abstract

Introduction

The objective of the pilot study was to determine the association between learning preferences and improvement in the American Academy of Neurology Residency In-Service Training Examination (RITE) scores from postgraduate year 2 (PGY-2) to postgraduate year 3 (PGY-3) in neurology residents.

Methods

Neurology residents at the University of Florida were approached to participate, and their consent was obtained. VARK inventory, representing four modalities (visual, aural, read/write, kinesthetic) of learning preferences, was completed by participants. Participants could pick more than one modality. The number of responses in each sensory domain was recorded, with higher numbers indicating stronger preference. Residents’ performance on the RITE was recorded for PGY-2 and PGY-3.

Results

Seventeen residents completed the VARK inventory and 16 had data for RITE. Residents demonstrated overall positive change in RITE from PGY-2 to PGY-3 (mean change = 6%; 95%CI: 4%, 9%). The median number of responses was highest for the kinesthetic domain (median = 7, range = 1-12), followed by visual (median = 6, range = 2-12), aural (median = 4, range = 1-10), and read/write (median = 4, range = 1-10). Among VARK domains, the number of responses in read/write had the strongest correlation with mean change in RITE performance from PGY-2 to PGY-3 (r = 0.45; 95%CI: -0.08, 0.78); residents in the high read/write group (number of response above median) had greater mean change in RITE performance (9%; 95%CI: 6%, 12%) while those in the low read/write group showed little to no increase in RITE from PGY-2 to PGY-3 (2%; 95%CI: -1%, 6%).

Conclusions

Higher VARK survey responses in the read/write domain were related to greater change in RITE scores from PGY-2 to PGY-3. These findings seem intuitively obvious considering the format of the RITE. These pilot data permit further investigation of individual resident learning preference and how it relates to test performance. By understanding a resident’s learning style, both educators and the resident will have an awareness of areas that need to be improved to be successful, which may be via remedial curricula and self-study activities.

## Introduction

Duty-hour restrictions limit the time available for resident education and decrease the amount of time with faculty [1]. However, medical care is constantly evolving, increasing the amount of information residents must learn. A clear strategy to improve teaching and learning efficiency would be beneficial. One potential strategy is to use resident learning styles, i.e., distinct preferences for receiving, processing, and assimilating knowledge [2].

The VARK model of learning styles was initially developed by Fleming in 1987 [2] and has been validated across a broad variety of learners [3,4]. VARK proposes that learning occurs via four sensory modalities: visual (V), aural (A), read/write (R), and kinesthetic (K). Associations between VARK modalities and resident achievement have been varied. Studies of surgical residents found positive relationships between visual, aural, and read/write modalities and scores on In-Training and USMLE examinations [5-7]. However, to the best of our knowledge, no study has examined the relationship between learning style preference and improvement on test scores as residents advance through their programs. Furthermore, little is known about the predominant learning preferences of neurology residents.

To better understand how learning strategies may impact neurology residents and before any strategies could be developed to apply learning styles to a neurology resident population, a pilot study was undertaken to determine the learning preferences within a neurology residency program. The primary objective was to determine if there is an association between VARK learning preferences and improvement in the American Academy of Neurology Residency In-Service Training Examination (RITE) scores from PGY-2 to PGY-3.

## Materials and methods

The study was approved by the University of Florida Institutional Review Board with consent obtained from all participants. Seventeen neurology residents from the University of Florida neurology residency in Gainesville, Florida, USA, agreed to participate and completed the VARK inventory. Participant demographic data collection included gender and postgraduate year level.

The VARK inventory is 16 multiple choice questions with four response options corresponding to one of four learning preference domains: visual, aural, read/write, or kinesthetic. The respondents are instructed to choose the answer that best explains their preference. If a single answer does not match their perception, more than one answer choice can be selected, and any question that does not apply can be left blank. The number of responses corresponding to each domain was recorded; higher numbers indicated a stronger preference for that learning modality. Based on this scoring, the participants were classified as having a predominant unimodal preference for visual, aural, read/write, or kinesthetic learning styles or a multimodal (MM) preference [2]. The MM category encompasses all possible combinations of two, three, or all four combinations of the sensory modalities. Resident performance on the RITE was recorded longitudinally for PGY-2 and PGY-3 years.

To examine the association between learning preferences and improvement in RITE score, two approaches were taken. First, Pearson correlations, with 95% confidence intervals, were estimated between the number of responses in each VARK domain and change in RITE scores (PGY-3 proportion correct - PGY-2 proportion correct). Next, the number of responses in each VARK domain was dichotomized into high vs. low (median split), calculated mean RITE change with 95% confidence interval for each group, with a similar approach for unimodal vs. MM preference. All analyses were performed in JMP Pro 15 (SAS Institute Inc., Cary, NC, USA).

## Results

Seventeen neurology residents at a single institution completed the VARK inventory for an overall response rate of 85% (out of 20 eligible residents), with 16 residents having available RITE scores at PGY-2 and PGY-3. Of the residents, four were women and seven had attained additional graduate or professional degrees in addition to their MD/DO degrees. Mean percent correct on RITE for PGY-2 and PGY-3 were 56% (SD = 9%) and 62% (SD = 10%), respectively. Residents demonstrated overall positive change from PGY-2 to PGY-3 (mean change = 6%, SD = 5%). Median number of responses was highest for the kinesthetic domain (median = 7, range = 1-12), followed by visual (median = 6, range = 2-12), with lower median responses for aural (median = 4, range = 1-10), and read/write (median = 4, range = 1-10) domains. Six of 17 (35%) residents had an MM learning style preference.

**Figure 1:**
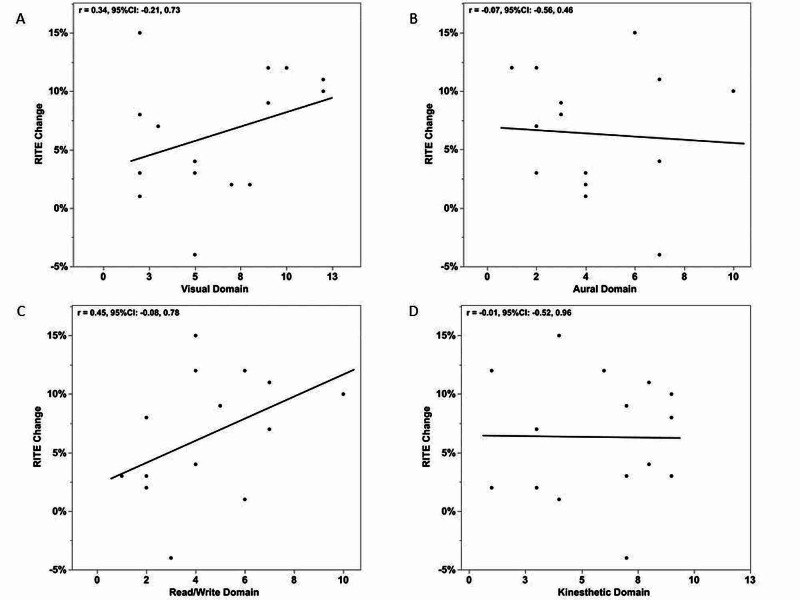
Correlations between the number of responses in each VARK domain and RITE improvement Pearson correlations between change (PGY3 – PGY2) in percent correct on Residency In-Service Training Examination (RITE) and the number of responses in each VARK domain: visual (A), aural (B), read/write (C), and kinesthetic (D). Correlations (r) reported with 95% confidence intervals (95%CI).

Figure 1 reports correlations between the number of responses in each VARK domain and the change in RITE between PGY-2 and PGY-3. Both read/write (Figure 1C) and visual (Figure 1A) domains had moderate, positive correlations with mean RITE change, suggesting greater responses in these domains may be associated with greater change on RITE performance from PGY-2 and PGY-3. However, the confidence intervals for each of these correlations did include zero.

**Figure 2:**
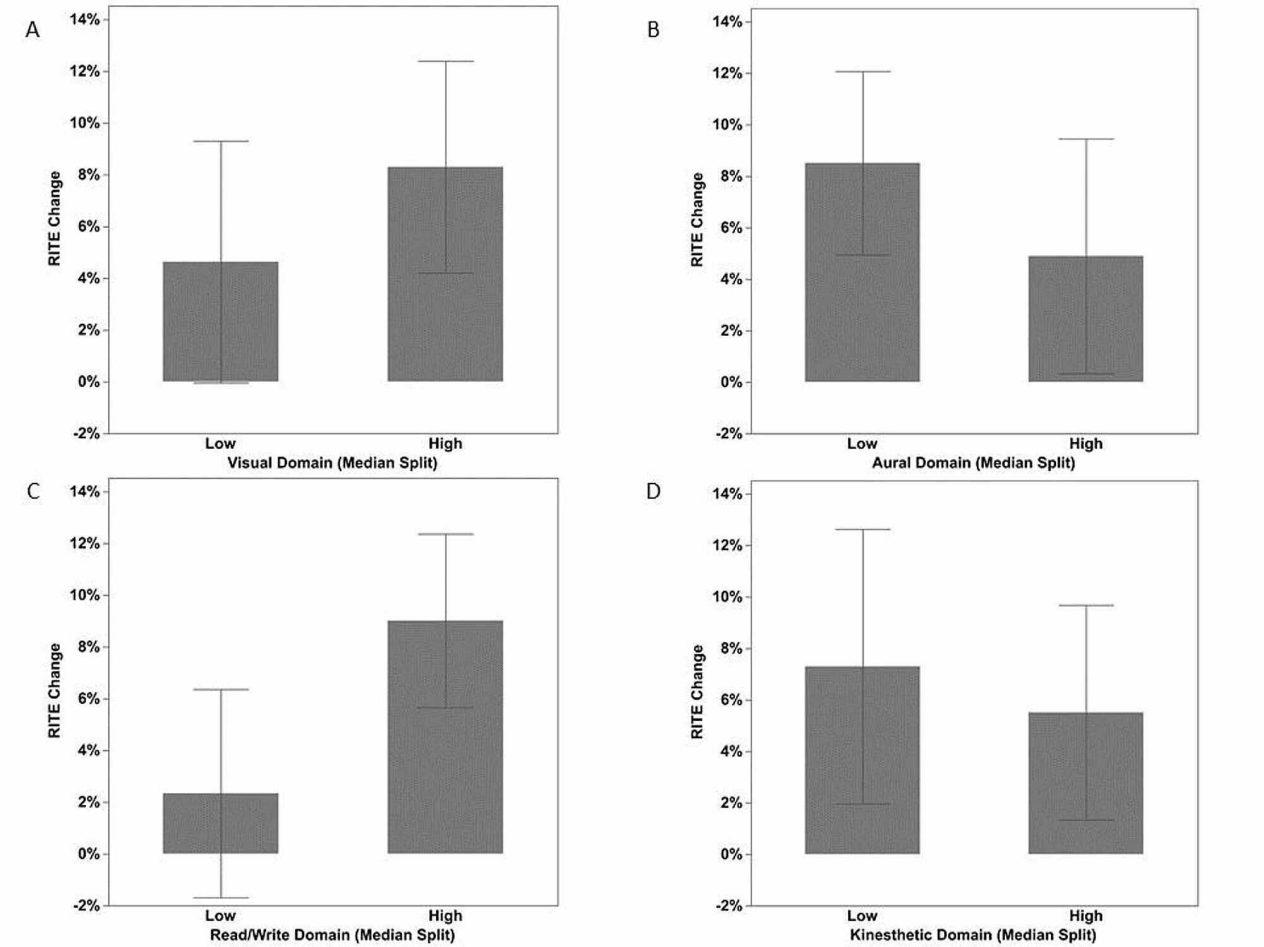
Mean change in RITE scores between median splits (high/low scores) for each domain Mean change (PGY3 – PGY2) in percent correct on Residency In-Service Training Examination (RITE) between median splits (high vs. low) for the number of responses in each VARK domain: visual (A), aural (B), read/write (C), and kinesthetic (D). Error bars are 95% confidence intervals.

Figure 2 displays the mean change in RITE scores between median splits (high/low scores) for each domain. Interestingly, when comparing mean RITE change in residents with high vs. low read/write responses (Figure 2C), residents having higher read/write preference had a larger mean change in RITE (9%; 95%CI: 6%, 12%). However, the low read/write group showed little to no change in RITE from PGY-2 to PGY-3 (2%; 95%CI: -1%, 6%), representing the worst performance of any of the domain groups. For MM preference, change in RITE was similar between unimodal (7%; 95%CI: 3%, 10%) and MM (6%; 95%CI: 1%, 11%) preferences.

## Discussion

The VARK inventory uses questions that address information processing in numerous situations so that learners are categorized by predominant learning styles [2]. This can be a unimodal learning preference (either visual, aural, read/write, or kinesthetic) or MM that represents a combination of these preferences. Visual and aural learners prefer to use these sensory modalities to learn, while read/write learners prefer reading and writing, and kinesthetic learners prefer interactive learning and using a “hands-on” approach.

The main goal of this study was to understand how learning preferences related to change in RITE scores from PGY-2 and PGY-3. To the best of our knowledge, this is the first study to evaluate how learning style preferences are associated with the change in performance over time in residents in general and a neurology resident cohort in particular. Higher numbers of responses for the read/write domain and, to a lesser extent, the visual domain were related to greater improvement in RITE scores. These findings seem intuitively obvious considering the format of the RITE, which is predominantly text and images. Thus, performance on the RITE and studying to improve performance would favor those with visual and read/write learning style preferences, and, conversely, not favor those who do not have these domain preferences. Our findings suggest the residents with low read/write domain preference showed the smallest improvement in RITE scores from PGY-2 to PGY-3.

In general surgery residents, the association between learning style preferences and test scores has varied. In one study, the VARK inventory number of aural responses was an independent predictor of higher scores on the American Board of Surgery In-Training Examination [5]. However, another study showed an association between higher scores and a dominant read/write preference [6]. Among general surgery resident interviewees, those with visual and aural dominant learning styles scored higher on the USMLE [7].

In the present study, the predominant learning preference for residents from a single neurology training program was kinesthetic, which is consistent with an interactive learning preference [5]. These neurology residents also showed a greater preference for visual learning. Six residents (35%) were MM. Based on a literature review at the time of the writing of this manuscript, this is the first time learning styles were evaluated in a cohort of neurology residents.

In a multi-institutional study examining VARK learning preferences of 132 general surgery residents across five surgery residency programs, the majority (61%) had an MM learning preference, with kinesthetic being the dominant unimodal preference (17%) [5]. In a department of pediatrics, 50 residents were assessed using the VARK inventory [8]. The VARK assessments demonstrated that 80% of those respondents were MM, with 90% having a kinesthetic learning preference. These previous studies, in combination with the present study, suggest there is heterogeneity in learning style preferences across specialties, which may be reflective of the heterogeneity in medical curricula and requirements of different departments.

Although, in theory, matching teaching techniques to the learners’ preferred learning style is beneficial, the actual impact of applying these strategies has been questioned [9]. The learners’ perceptions of benefits from how their education is presented are important; however, the learner perception and the actual demonstrated attainment of knowledge are often inconsistent [10]. Thus, understanding learning styles and the impact of the curriculum when applied to the learning style preferences requires further investigation. Overall, as medical training progresses, it has been shown that learning styles and preferences may be adopted by individuals to their curriculum and types of examinations [11,12]. With the RITE specifically and clinical training overall, all modalities need to be refined in order to achieve career success. By understanding a resident’s learning style, both educators and the resident will be aware of areas that need to be improved to be successful, which may be via remedial curricula and self-study activities [6].

There are limitations to this study. This study represents a pilot study of a single neurology training program in a university-based program with a small sample size, which may not be representative of neurology residents as a whole and was underpowered to detect statistically significant relationships. This study was limited to evaluating RITE scores in representing the core knowledge during residency. Additionally, other measures of resident performance were not evaluated, including assessment of technical and non-technical skills and faculty evaluations; VARK learning preferences may have different associations with these measures. Finally, other factors, out of the scope of this study, may affect RITE performance and change in RITE across residency.

## Conclusions

This pilot study demonstrated that the most common VARK inventory learning preference of one neurology residency training program was kinesthetic, followed by sensory and visual. However, higher read/write responses were related to greater RITE score improvement in this group. These pilot data permit further investigation of individual resident learning preferences and the resident's perceived effectiveness to varying approaches to presenting curriculum material. This information will hopefully better enable educators to understand the importance of learning preference styles when evaluating the curriculum and instituting programmatic improvements. Further evaluation is also required to determine the overall impact of the individual learning preference on neurology resident education.
